# The effects of raloxifene treatment on oxidative status in brain tissues and learning process of ovariectomized rats 

**Published:** 2011

**Authors:** Süreyya Osmanova, Ebru Sezer, Volkan Turan, Burak Zeybek, Mustafa Cosan Terek, Lutfiye Kanıt

**Affiliations:** 1 Department of Obstetrics and Gynecology, Faculty of Medicine, Ege University, Bornova, Izmir, Turkey.; 2 Department of Biochemistry, Ege University, Faculty of Medicine, Bornova, Izmir, Turkey.; 3 Department of Physiology, Ege University, Faculty of Medicine, Bornova, Izmir, Turkey.

**Keywords:** *Oxidative stress*, *Raloxifene*, *Active avoidence task*, *Cognitive process*

## Abstract

**Background:**

The effects of estrogene on central nervous system are still controversial.

**Objective:**

We aimed to investigate the effects of raloxifene on the antioxidant enzyme [superoxide dismutase (SOD) and catalase (CAT)] activities and malondialdehyde (MDA) levels in brain homogenates of ovariectomized female rats and its effect on cognitive process of learning.

**Materials and Methods:**

Female Sprague Dawley rats (n=24) were divided into three groups. Three weeks after ovariectomy; nonovariectomized group (control group) (n=8) was given physiological saline (SP) as placebo. First ovariectomized group (n=8) received raloxifene 1mg/kg dissolved in a 1% solution of carboxymethylcellulose (CMC) subcutaneusly (sc) and second group of ovariectomized rats were given 1 % CMC 1mg/kg (sc) every day for 14 days. Learning behaviors of rats were evaluated in active avoidence cage with using sound and electrical stimulation. The levels of oxidative stress (MDA) and antioxidant enzymes (SOD, CAT) in different regions of the brain homogenates were compared between three groups of decapitated rats.

**Results:**

Raloxifene had a significant attenuating effect on the levels of MDA in brain tissues suggesting raloxifene’s effect against lipid peroxidation at the end of training days. With the comparison of brain regions, cortex showed the highest average activity of SOD and CAT and cerebellum had the lowest average levels for both. Its effects on learning and cognitive process with active avoidence task were considered insignificant.

**Conclusion:**

Raloxifene treatment may have preventive effects for the brain against oxidative stress and lipid peroxidation in rats.

## Introduction

Selective estrogen receptor modulators (SERMs) are compounds that act as estrogen agonists on selected targets (bone and brain) while being estrogen antagonists on others (breast and uterus) (1). Recent studies suggest that raloxifene has neuroprotective action in the central nervous system and demonstrates a pharmacological profile similar to that of 17 -estradiol (E_2_) in both ovariectomized rats and postmenopausal women (2). Most degenerative diseases are a concequence of repeated oxidative levels (3, 4). 

The incidence of most degenerative diseases including cancer, cardiovasculer diseases, cataract and Alzheimer’s disease increase by aging, supporting the role of repeated oxidative damage in the pathophysiology of these medical conditions. Several methods have been used to assess the level of oxidative damage in human body. 

Malondialdehyde (MDA) is one of lipid peroxidation product that can be used as a marker of oxidative stress. Natural antioxidant enzymes have an important role against free radicals in human body. Glutathione peroxidase, glutathione reductase, catalase (CAT), thioredoxin reductase, superoxide dismutase (SOD), and heme oxygenase are some of the most important antioxidant enzymes. The enzyme superoxide dismutase converts two superoxide radicals into one hydrogen peroxide and one oxygen. Catalase catalyses conversion of hydrogen peroxide, a powerful and potentially harmful oxidizing agent to water and molecular oxygen (5). 

To explain how people acquire skills, knowledge, and attitudes, learning theory was described. Various branches of learning theory are used to improve and accelerate the learning process including social facilitation, observation, formal teaching, memory, mimicry, and classical and operant conditioning. *Classical* conditioning forms an association between two stimuli. *Operant *conditioning forms an association between a behavior and a consequence (6). 

There were studies about the prevention loss of cognitive function and improvement of oxidative stress subsequent to raloxifene treatment (2, 7, 8). Different results obtained in these studies. The aim of present study was to investigate the effects of raloxifene on the antioxidant enzyme (superoxide dismutase and catalase) activities and malondialdehyde levels in brain homogenates of ovariectomized female rats and its effect on cognitive process of learning. 

## Materials and methods

**Animals and treatments**

Adult female Sprague-Dawley rats weighting 300-350 gram that models the human condition, used for this prospective randomized study. The protocol for the experiment was approved by the Appropriate Animal Care Committee of Ege University. 

To reduce circulating 17 -estradiol levels, 16 animals were bilaterally ovariectomized under pentobarbital sodium anastesia when they were four months old. Ovariectomized animals were divided into two groups, three weeks after ovariectomy. All animals (n=24) were divided into three experimental groups:

1) Non ovariectomized group (n=8) was used as a naive controls with the treatment of physiological saline (SP) 1 mg/ kg subcutaneously daily every morning at 09:00 am for 14 days, 

**2) Ovariectomized group (n=8) was given drug vehicle (% solution of carboxy methyl cellulose) 1 ml/ kg subcutaneously daily every morning at 09:00 am for 14 days,**

3) Raloxifene group (n=8) was given raloxifene 1 mg/ kg subcutaneously (dissolved in a %1 solution of carboxy methyl cellulose) daily every morning at 09:00 am for 14 days, 

At the end of the 12^th^ day learning experiments were started in active avoidence cage. The treatments were carried out for two days at the same time with learning process and stopped on the 14^th^ day.

**Active avoidence experiment**

The mouse has to learn to escape from a specific stimulus, by actively moving to a different compartment. The number of avoidances (the mouse passing to the other compartment during the stimulus signal), number of non-response, response latency (latency to escape) were recorded. These measures serve as an index of learning and allows memory to be evaluated. 

We used sound stimulation for 3 seconds (78 dB, 380 Hz) and waited for 5 seconds. After this, we applied 50 volt electrical footshock for 3 seconds. As soon as the sound was heard by the animal, climbing the wood bar without taking any electrical shock was accepted as a true behavior (learning). Experiments were done for 5 days with the number of 15 training per day. 

**The collection of tissue samples and preparation**

At the end of the training days rats were decapitated and brain samples were dissected on ice by the experienced investigators. Rat brain tissues were dissected to cortex, striatum, hippocampus and cerebellum regions. 

All samples were stored at -80˚C until they were used. Samples were weighed and 0.2-0.6 g samples were homogenized with phosphate- buffered saline (PBS, 0.01 M pH: 7.4). The homogenates were centrifuged for 15 minutes at 10000 g and the supernatants were collected and kept frozen at -80˚C for measurement of antioxidant enzyme activities and MDA. Catalase activity was determined as described by Sözmen *et al* (9) in which the degradation of hydrogen peroxide is recorded spectrophotometrically at 240 nm. One unit of CAT was defined as the amount of enzyme, which decomposes 1 µmol H_2_O_2_/min under specific conditions. The results were expressed as U/gr protein. 

Superoxide dismutase activity was measured by colorimetric method based on the inhibition of autoxidation epinephrine by SOD at 480 nm. The results were expressed as U/mg protein. MDA was measured by TBA method spectrophotometrically. After adding TBA, homogenates were boiled at 100˚C for 20 minutes and centrifuged for 10 minutes. After being cooled, supernatant was measured at 532 nm colometrically. The results were expressed as nmol/mg tissue.

**Statistical analysis**

Experimental results were analysed by the Mann Whitney U test (SPSS for Windows release 11.0). Kruskal- Wallis test was also used for the comparison of more than one means of non-parametrical data. Difference were considered significant at p< 0.05 level. 

## Results

SOD and CAT enzyme activities in different regions of brain of non-ovariectomized, ovariectomized and raloxifene groups are given in table I, II and table III respectively. MDA levels in the brain tissues are presented in table IV. Comparison of non-overectomized SP group, overectomized CMC group and overectomized raloxifene treated group showed that raloxifene treatment caused a significant decrease in levels of MDA in whole brain tissue. (Table I) and also for each region of brain studied separately (Table IV). Yet there was no significant difference in SOD and CAT activities between groups (Table II, III). When we compare all groups for different regions of brain the following results were achieved: Evaluation of brain regions’ enzyme activities with each other showed that cortex had significantly higher SOD and CAT activity in comparison to striatum, hippocampus and cerebellum regions (p<0.05). 

**Figure 1:**
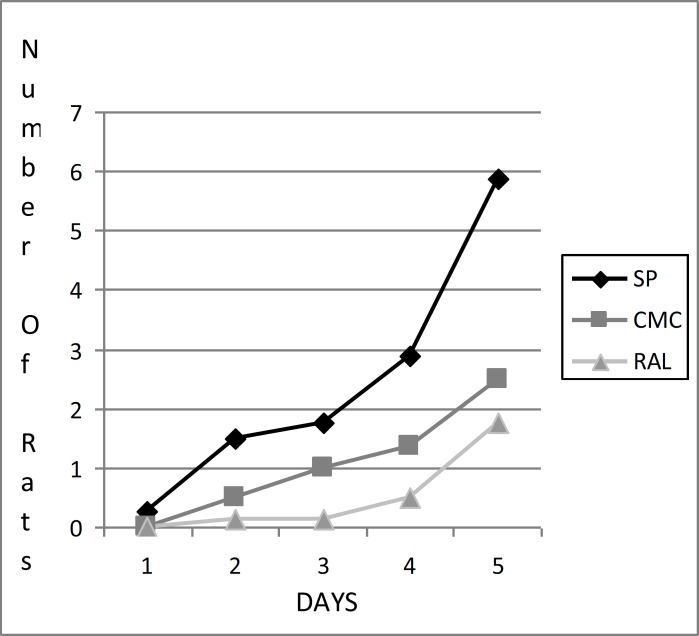
The results of training on active avoidence behavior for 5 days. (SP: , Physiological saline CMC: Carboxy methyl cellulose RAL: raloxifene ).

There was no significant difference between MDA levels. The striatum and hippocampus showed no difference in respect SOD activity, CAT and MDA levels. SOD activity was found significantly different between striatum and cerebellum (p<0.001). Between hippocampus and cerebellum SOD and CAT activities showed significant difference (p=0.008, p=0.002) but there was no significant difference for MDA levels were (p=0.902). While cortex has the highest average levels of SOD and CAT, cerebellum has the lowest average levels for both in all groups. The results of learning avoidance experiments showed no statistical significance among three groups (Figure 1). At the end of fifth day SP group showed the highest learning activity. 

**Table I:** Average levels of superoxide dismutase, catalase and malondialdehyde for each three groups (* p< 0,05) Values are given as mean±SD.

	**Superoxide-dismutase** **(U/mgr. protein)**	**Catalase** **(U/mgr. protein)**	**Malondialdehyde** **(nmol/mgr. protein)**
Physiological saline group	4.66± 0.96	0.58 ± 0.14	19.69 ± 1.66
Carboxymethylcellulose group	4.44 ± 1.08	0.64±0.17	19.56 ± 3.15
Raloxifene group	4.26 ± 0.80	0.60 ± 0.17	13.07 ± 1.84*

**Table II:** Superoxide dismutase levels for different brain regions. (Values are given as mean±SD, difference were considered significant at p<0.05 level).

**Brain regions**	**Physiological saline** **(nmol/mg. protein)**	**Carboxy Methyl cellulose** **(nmol/mg. protein)**	**Raloxifene** **(nmol/mg. protein)**	**p-value**
Cortex	14.81 ± 1.71	20.16 ± 3.21	13.66 ± 1.67	0.001
Striatum	13.84 ± 1.05	18.78 ± 2.29	12.37 ± 1.73	0.001
Hippocampus	15.36 ± 1.91	19.59 ± 3.01	13.26 ± 1.58	0.002
Cerebellum	15.60 ± 1.55	19.74 ± 4.28	12.98 ± 2.39	0.007

**Table III:** Catalase levels for different brain regions. (Values are given as mean±SD, Difference were considered significant at p<0.05 level).

**Brain regions**	**Physiological saline** **(U/mg. protein)**	**Carboxy methyl cellulose** **(U/mg. protein)**	**Raloxifene** **(U/mg. protein)**	**p-value**
Cortex	0.70±0.83	0.70±0.19	0.69±0.19	0.867
Striatum	0.52±1.27	0.64±0.14	0.54±0.14	0.142
Hippocampus	0.61±0.12	0.70±0.16	0.64±0.19	0.445
Cerebellum	0.51±0.62	0.52±1.46	0.52±0.12	0.911

**Table IV:** Malondialdehyde levels for different brain regions. Raloxifene is effective in all regions of brain for reducing lipid peroxidation (Values are given as mean± SD, Difference were considered significant at p<0.05 level ).

**Brain regions**	**Physiological saline** **(U/mg.protein)**	**Carboxy methyl cellulose** **(U/mg.protein)**	**Raloxifene** **(U/mg.protein)**	**p-value**
Cortex	4.57 ± 0.83	5.64 ± 0.65	5.08 ± 0.54	0.251
Striatum	3.93 ± 0.52	4.49 ± 0.62	4.31 ± 0.42	0.163
Hippocampus	3.91 ± 0.36	4.31 ± 0.68	3.99 ± 0.80	0.348
Cerebellum	3.51 ± 0.45	3.31 ± 0.89	3.67 ± 0.68	0.733

## Discussion

Estrogens exert profound effects on growth, differentation and function of many reproductive tissues. They also effect other tissues, including bone, liver, cardiovasculer system and brain. As women undergo menopause, circulating concentrations of estrogen decrease. 

The relative estrogen deprivation in postmenopausal women is associated with physiological changes and an increased level of reactive oxygen species and the risk of several diseases including cardiovasculer disease, osteoporosis and degenerative processes in the central nervous system (9, 10). It has been reported that oxidative stress enhanced in female rat brain after gonadectomy and 17ß-estradiol protected the hippocampus, cerebral cortex and the hypotalamus from chronic stress (11, 12). 

Recent studies suggested that estrogen may be used to protect Alzheimer Disease development as well as using for the prevention of osteoporosis and cardiovasculer disease (13, 14). Raloxifene is a tissue- selective estrogen receptor modulator. While estrogenic effects were utilised on bone and lipid metabolism; on endometrium and breast tissue, it acted as antiestrogenic. Therefore raloxifene seems to be more advantageus with fewer side effects than estrogen therapy. 

Animal studies and invitro investigations have shown antioxidant and vasoprotective effects of raloxifene (15, 16). A multicenter, randomized, double- blind trial, included postmenopausal women with osteoporosis to investigate the safety and adverse effects associated with raloxifene. It was found to be associated with an increased risk for venous thromboembolism. 

But there was no increased risk for endometrial hyperplasia or endometrial cancer which were at risk in estrogen therapy (17, 18). Haskell *et al* (7) reported the effect of estrogen replacement therapy on cognitive function in women. Nineteen studies were reviewed, including 10 randomized trials of estrogen replacement therapy versus placebo. Baseline characteristics were evaluated including age, menopausal status, follicle-stimulating hormone, luteinizing hormone, and estradiol levels, mood and measures of cognitive function.

Of the 10 randomized trials, eight claimed therapeutic benefits for estrogen therapy, three of which reported significant improvements in memory and two of which showed improvements in attention. These studies did not control for potential confounds such as depression and vasomotor symptoms. Gibbs (19) and Henderson and Brintor (20) suggested there is strong and consistent evidence of neuroprotective effects of estrogen in animal models unlike Barrett Connor *et al* (8). who determined whether replacement estrogen delays or prevents loss of cognitive function in elderly women in a prospective study that lasts 15 years and reported no effect of estrogen on cognitive function was shown in these older women.

Topcuoglu *et al* (21) assessed estrogen-related changes in the redox status of the brain and liver proteins as well as the systemic oxidative stress in ovariectomized rats and showed the extent of oxidative protein damage (OPD) in this model. At the end of the study they claimed that estrogens played an important role within the antioxidant defense systems in plasma, liver and brain by decreasing oxidative stress.

Ghidoni *et al* (22) documented the effects of estrogens on cognition and brain morphology. Estrogen use appeared to improve linguistic, attentive and planning abilities. The beneficial effects on cognition were detected mainly in the past users subgroup**.** Ozgonul *et al* (23) investigated the effects of estradiol and raloxifene on antioxidative enzyme status and MDA levels in brain and liver homogenates of ovariectomized female rats. 

Raloxifene treatment was not found as effective as estrogen therapy on catalase activity. Superoxide dismutase activities and MDA levels in liver did not change in all groups. No significancy was considered in the brain tissue SOD and CAT activities between the control ovariectomy, estrogen treated, and raloxifen treated groups while raloxifene treatment decreased MDA to normal limits. 

They suggested estrogen and raloxifene therapy is more effective in brain rather than liver and reported raloxifene can be suggested primarily for treatment and prevention of diseases caused by oxidative stress in postmenopausal women. Konyalıoglu *et al* (24) performed similar study with ovariectomized rats that were given raloxifene 1 mg/ kg sc daily for 12 days and demonstrated that raloxifene may be more effective against oxidative stress in heart and brain than in liver tissue.

In our study, raloxifene demonstrated no significant changes on anti- oxidant enzymes activities although it had a significant attenuating effect on the levels of MDA. Its effects on learning and cognitive process with active avoidence task were considered insignificant. 

In conclusion, estrogen and estrogen receptor modulators have been widely used for postmenopausal hormone replacement therapy and in vitro studies suggested that they may exhibit pro- and anti-oxidant effects in a dose and tissue dependent manner. Although usage of raloxifene in a short period seems to be ineffective on cognitive process in this study, long term studies are required. Future studies may focused on different types of treatment for brain activities in postmenapausal women. 

## Confilict of intrest

There is no conflict of interest.
